# Assessment of radiation sensitivity of unresectable intrahepatic cholangiocarcinoma in a series of patients submitted to radioembolization with yttrium-90 resin microspheres

**DOI:** 10.1038/s41598-021-99219-7

**Published:** 2021-10-05

**Authors:** Tommaso Depalo, Antonio Claudio Traino, Irene Bargellini, Giulia Lorenzoni, Elena Bozzi, Caterina Vivaldi, Rocco Lamastra, Gianluca Masi, Roberto Cioni, Giuseppe Boni, Duccio Volterrani

**Affiliations:** 1 Regional Center of Nuclear Medicine, University Hospital of Pisa, Pisa, Italy; 2 Health Physics Unit, University Hospital of Pisa, Pisa, Italy; 3 Department of Vascular and Interventional Radiology, University Hospital of Pisa, Pisa, Italy; 4 Division of Medical Oncology, University Hospital of Pisa, Pisa, Italy

**Keywords:** Cancer, Oncology

## Abstract

Radioembolization is a valuable therapeutic option in patients with unresectable intrahepatic cholangiocarcinoma. The essential implementation of the absorbed dose calculation methods should take into account also the specific tumor radiosensitivity, expressed by the α parameter. Purpose of this study was to retrospectively calculate it in a series of patients with unresectable intrahepatic cholangiocarcinoma submitted to radioembolization. Twenty-one therapeutic procedures in 15 patients were analysed. Tumor absorbed doses were calculated processing the post-therapeutic ^90^Y-PET/CT images and the pre-treatment contrast-enhanced CT scans. Tumor absorbed dose and pre- and post-treatment tumor volumes were used to calculate α and α_3D_ parameters (dividing targeted liver in n voxels of the same volume with specific voxel absorbed dose). A tumor volume reduction was observed after treatment. The median of tumor average absorbed dose was 93 Gy (95% CI 81–119) and its correlation with the residual tumor mass was statistically significant. The median of α and α_3D_ parameters was 0.005 Gy^−1^ (95% CI 0.004–0.008) and 0.007 Gy^−1^ (95% CI 0.005–0.015), respectively. Multivariate analysis showed tumor volume and tumor absorbed dose as significant predictors of the time to tumor progression. The knowledge of radiobiological parameters gives the possibility to decide the administered activity in order to improve the outcome of the treatment.

## Introduction

Loco-regional therapies represent a valid alternative to systemic therapy in primary liver malignancy with no or limited extrahepatic disease. Among them, selective internal radiation therapy (SIRT) has been advocated as a well-tolerated and effective therapeutic approach consisting of the intra-arterial injection of micron-sized particles loaded with yttrium-90 (^90^Y)^1,2^.

Routinely, administered activity is established by the body surface area-based (BSA) method^3^ for resin microspheres (SIR-Spheres, Sirtex Medical Products, Sydney, Australia) but it often results in underdosing (and in some cases overdosing) treatments^4–6^.

Pre-treatment SPECT-CT (based on 99m-technectium macroaggregates of albumin; ^99m^Tc-MAA) and post-treatment ^90^Y-PET-CT^7,8^ allow to perform 3D dosimetry taking into account the 3D distribution of ^90^Y activity within the targeted liver and tumor. This approach is more effective than the BSA method^9–11^.

Although the improvement of absorbed dose calculation method could have a direct impact on the clinical outcome, variable radiosensitivity of the different tumors and hepatic tissue in SIRT should also be taken into account^12^. The radiosensitivity is expressed by the tumor-specific radiobiological parameter α that represents the response of the treated tissue to the irradiation.

Based on the well-known Linear-Quadratic (LQ) radiobiological model^13,14^, the knowledge of α could help to improve SIRT planning personalized activity in order to reach better clinical outcomes.

Purpose of this retrospective study was to calculate the radiobiological α parameter in a series of patients with unresectable intrahepatic cholangiocarcinoma (ICC) submitted to SIRT with ^90^Y resin microspheres.

## Materials and methods

### Radiobiology: theoretical aspects

The well-known LQ model represents the main equation of radiobiology:

1 $$N={N}_{0}{e}^{-\left(\alpha D+G\beta {D}^{2}\right)}$$

with N_0_ the number of tumor cells before the treatment; N the number of tumor cells remaining after treatment; D the average tumor absorbed dose; α and β the so-called intrinsic radiosensitivity; G the Lea-Catcheside factor, taking into account the capability of the cells to self-repair themselves.

A reasonable simplification for radioembolization (low dose-rate) is to neglect quadratic effects (i.e., Gβ = 0):

2 $$N={N}_{0}{ e}^{-\alpha D}$$

Assuming a linear relationship between the number of tumor cells and the macroscopical mass of the lesion (M) the simplified LQ model (Eq. 2) becomes:

3 $${M}_{f}={M}_{0} {e}^{-\alpha D}$$

with M_f_ the minimum value of post-treatment tumor mass and M_0_ the pre-treatment tumor mass.

In Eq. (3), α is the only unknown parameter: in fact, M_f_ and M_0_ can be measured and D can be easily calculated. Note that the knowledge of α allows to predict the best response M_f_ to the therapy.

From Eq. (3), α can be calculated as:

4 $$\alpha =-\frac{1}{D}\mathrm{ln}\left(\frac{{M}_{f}}{{M}_{0}}\right)$$

In SIRT, the distribution of the activity in the targeted liver cannot be considered homogeneous. Dividing the targeted liver in n voxels of the same volume (m_0_), Eq. (3) becomes:

5 $${M}_{f}= {m}_{0}\sum_{i=1}^{n}{e}^{-{\alpha }_{3D}{d}_{i}}$$

with d_i_ the i-th voxel absorbed dose. Again, it is possible to evaluate α_3D_ by numerically solving Eq. (5).

### Study participants and study design

This retrospective study enrolled twenty-six patients with histologically-proven unresectable ICC, who referred to our center for ^90^Y-SIRT from July 2013 to June 2018. Eight patients were excluded because of unavailability of post-therapy ^90^Y-PET/CT (n = 4) or post-treatment radiological follow-up (n = 4). Moreover, 3 patients with disease progression at the first radiological follow-up were excluded because of a very low average tumor absorbed dose (< 35 Gy).

The clinical data of the 15 patients included in the study are summarized in Table 1. Patients were indicated to SIRT after multidisciplinary tumor board discussion. In 7 patients (47%), SIRT was associated to systemic chemotherapy as consolidation therapy.

**Table 1:** Baseline clinical characteristics.

Characteristic		Value
Gender	Male	10 (67)
	Female	5 (33)
Age at diagnosis (years)	Median (95% CI)	61 (56–66)
BSA (m^2^)	Median (95% CI)	1.8 (1.7–1.9)
Time between diagnosis and SIRT (months)	Median (95% CI)	12.2 (6.9–25.3)
ECOG status	0	13 (87)
	1	2 (13)
Liver cirrhosis	Yes	4 (27)
Child–Pugh score	A5	12 (80)
	A6	3 (20)
TNM stage	III	6 (40)
	IVa	3 (20)
	IVb	6 (40)
Pre-treatment Ca19.9 (UI/l)	Median (95% CI)	14.1 (10.9–62.9)
Tumor presentation	Solitary	9 (60)
	Multifocal	6 (40)
Tumor distribution	Unilobar	10 (67)
	Bilobar (mass-forming)	3 (20)
	Bilobar (multifocal)	2 (13)
Tumor infiltration		9 (60)
Portal vein thrombosis	Yes	5 (33)
Naïve to treatment	Yes	6 (40)
Previous treatment	Yes	9 (60)
	Surgery	5 (33)
	Chemotherapy	9 (60)
	Radiofrequency ablation	1 (7)
	External beam radiotherapy	1 (7)
Chemotherapy associated to SIRT	Yes	7 (47)

When not otherwise specified data are expressed as numbers (percentages).

*BSA* body surface area, *SIRT* selective internal radiation therapy, *ICC* intrahepatic cholangiocarcinoma.

Five out of the 15 patients selected for α calculation had bilobar disease treated in two different sessions within 4 weeks (3 patients presented mass-forming lesions, whereas 2 patients were characterized by multifocal lesions); in one patient treatment was repeated on the same lesion one year after the first treatment; thus, overall 21 SIRT procedures were performed in the selected population. Since it was not possible to obtain two separated values of M_f_ in patients with bilobar mass-forming lesions, the data of two separate SIRT procedures performed in these patients were summed up to calculate the α values from Eq. (4), resulting in 18 α values. For the measurement of the α_3D_ values from Eq. (5), the data of SIRT procedures performed in mass-forming lesions were excluded, resulting in 15 α_3D_ values.

### SIRT procedure

All patients had a preliminary angiography, followed by the intra-arterial injection of ^99m^Tc-MAA (Technescan LyoMAA, Mallinckrodt Medical, Petten, The Netherlands) with an activity of about 185 MBq, at the selected injection site. Then, patients underwent a total body planar acquisition and SPECT/CT of upper abdomen by means of a hybrid scanner DiscoveryNM/CT670 (GE Healthcare, Waukesha, WI, USA) with 16-slices CT BrightSpeed Elite. SPECT/CT acquisitions were conducted using the following parameters: window 140 ± 7.5 keV; 120 projections for an orbit of 360°; matrix 128 × 128; 10 s/projection. Data were reconstructed using an iterative algorithm (OSEM, 2 iterations, 8 subsets) with CT based attenuation and scatter corrections.

In the absence of clinically relevant lung shunt fraction (LSF < 20%) and abdominal extra-hepatic shunts, patients underwent SIRT within 21 days, injecting ^90^Y resin microspheres at the level of the planned injection site. In case of bilobar tumors (5/15), patients were treated with two subsequent procedures, at an interval of approximately 4 weeks.

The administered activity was determined by using the BSA method. The volumes of total liver, targeted liver and tumor were obtained on pre-treatment contrast-enhanced CT by an implemented software using a semi-automatic contouring on a dedicated workstation (Advantage Window 4.7, GE Healthcare). Considering the liver/tumor tissue density as 1 g/mL, the liver/tumor mass was calculated from liver/tumor volume.

### Post-treatment data and outcomes

Within 15 h from the SIRT procedure, patients performed post-therapeutic ^90^Y-PET/CT scan of upper abdomen with a hybrid scanner Discovery710 (GE Healthcare Milwaukee, Wisconsin, USA) equipped with a 64-slices Optima CT660. PET acquisitions were obtained including the whole liver lasting 20 min/bed (usually 2 beds for each acquisition). Vue Point FX algorithm including Time-of-Flight information was used for reconstruction (2 iterations, 16 subsets). The ^90^Y-PET/CT was useful to confirm the hepatic distribution of microspheres and to calculate the average absorbed dose and the 3D distribution of the absorbed dose within each tumor.

**Figure 1:**
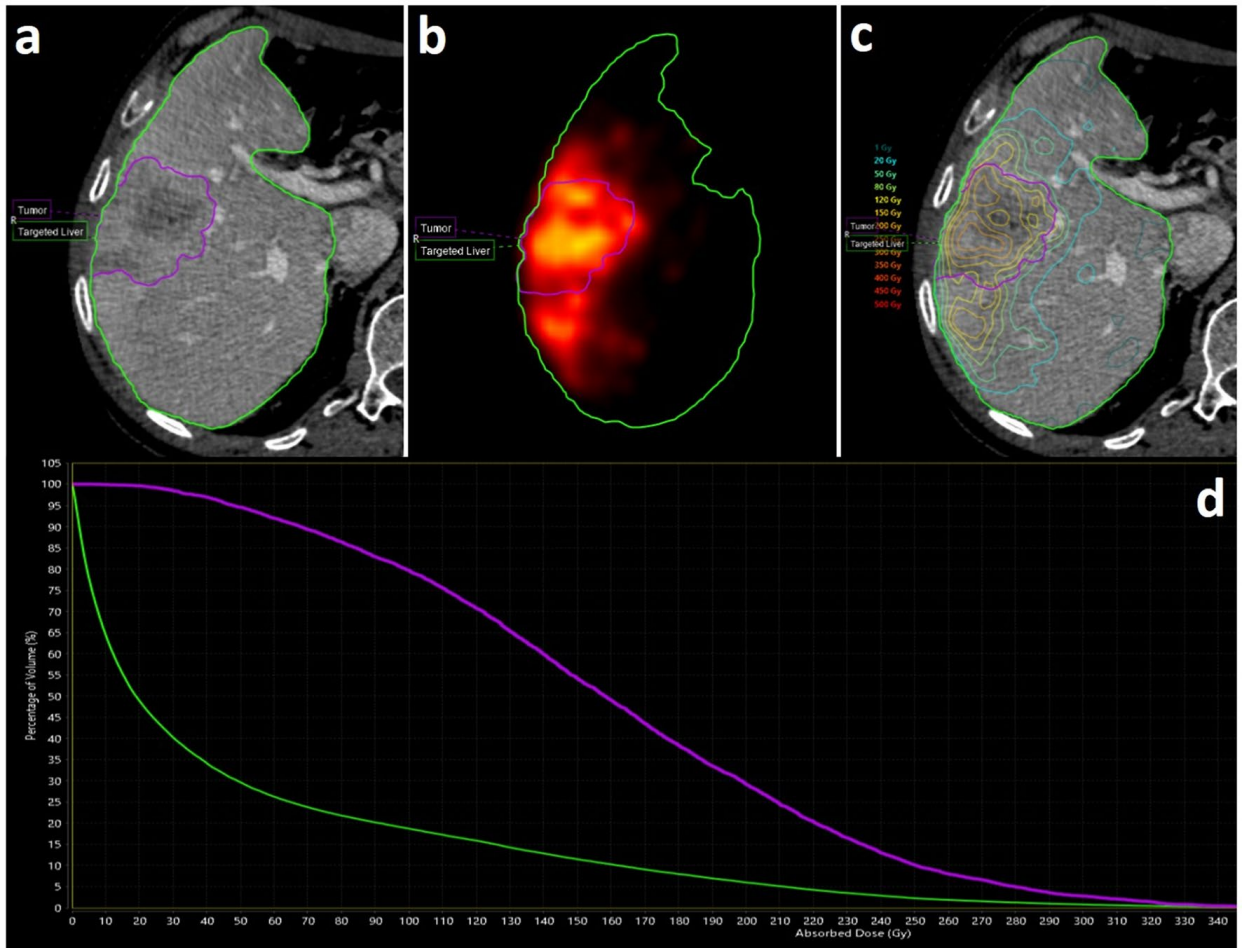
A representation of imaging processing by using the dosimetry software (Simplicit90Y, Mirada Medical, Oxford, UK). On upper left panel (**a**), VOIs semi-automatically defined on the contrast-enhanced CT over the targeted liver and tumor. On upper central panel (**b**), the same VOIs transferred over the previously co-registered ^90^Y-PET. On upper right panel (**c**), calculated isodose distribution within the targeted liver and tumor. On bottom panel (**d**), dose-volume histograms (DVH) of the targeted liver and tumor.

^90^Y-PET/CT and contrast-enhanced CT scans were processed by using a dosimetry software (Simplicit90Y, Mirada Medical, Oxford, UK). Volumes of interest (VOIs) were semi-automatically defined on the contrast-enhanced CT over the total liver, targeted liver and tumor, and transferred on the corresponding PET slices after the co-registration between the contrast-enhanced CT and the low-dose CT of the PET/CT scan. After entering the LSF values and the actual administered activity, the software turns out the dose-volume histograms (DVH) and the values of average absorbed dose of the targeted liver and tumor (Fig. 1).

The minimum dose absorbed by the 70% of tumor volume (D70) and the percentage of tumor volume that absorbed at least 100 Gy (V100) were extrapolated from the DVHs, as good predictors of therapeutic efficacy^15,16^.

During the follow-up, patients underwent contrast-enhanced CT scan 4–6 weeks after treatment and then every 3 months, to evaluate radiological tumor response according to the Response Criteria in Solid Tumors (RECIST) 1.1 criteria. The minimal mass of the treated tumor (M_f_), corresponding to the maximum reduction of M_0_ after SIRT, was evaluated for each treatment on the contrast-enhanced CT scans, using the same method employed to obtain M_0_.

### Statistical analysis

Quantitative values were expressed in terms of median in association of 95% confidence interval (95% CI). The non-parametric Wilcoxon comparison test was used to calculate differences between repeated measurements, before and after treatment. Correlations between absorbed dose measurements and tumor volume changes were performed.

Time to progression (TTP) was calculated as the time between first SIRT and radiological tumor progression, using the Kaplan–Meier method. Tumor-related continuous variables were analyzed for an association with TTP by univariate and multivariate Cox regression models and the log-rank test. Variables with p < 0.1 in the univariate analysis were included in the multivariate analysis model.

JMP8 software (Statistical Discovery ™) was used for statistical analysis.

### Ethics approval

All procedures performed in studies involving human participants were in accordance with the ethical standards of the institutional and/or national research committee and with the 1964 Helsinki declaration and its later amendments or comparable ethical standards. This study was approved and informed consent was waived by the Ethics Committee for the Vast North West Area (CEAVNO 19/11/2020)—Tuscany, Italy. This article does not contain any studies with animals performed by any of the authors.

## Results

Considering the 21 SIRT procedures, LSF was ≤ 6% and the administered activity of ^90^Y microspheres was 1166 MBq (95% CI 926–1316). Considering the density of liver tissue as 1 g/mL, targeted liver mass and average absorbed dose were 1260 g (95% CI 990–1501) and 42 Gy (95% CI 39–51), respectively.

**Figure 2:**
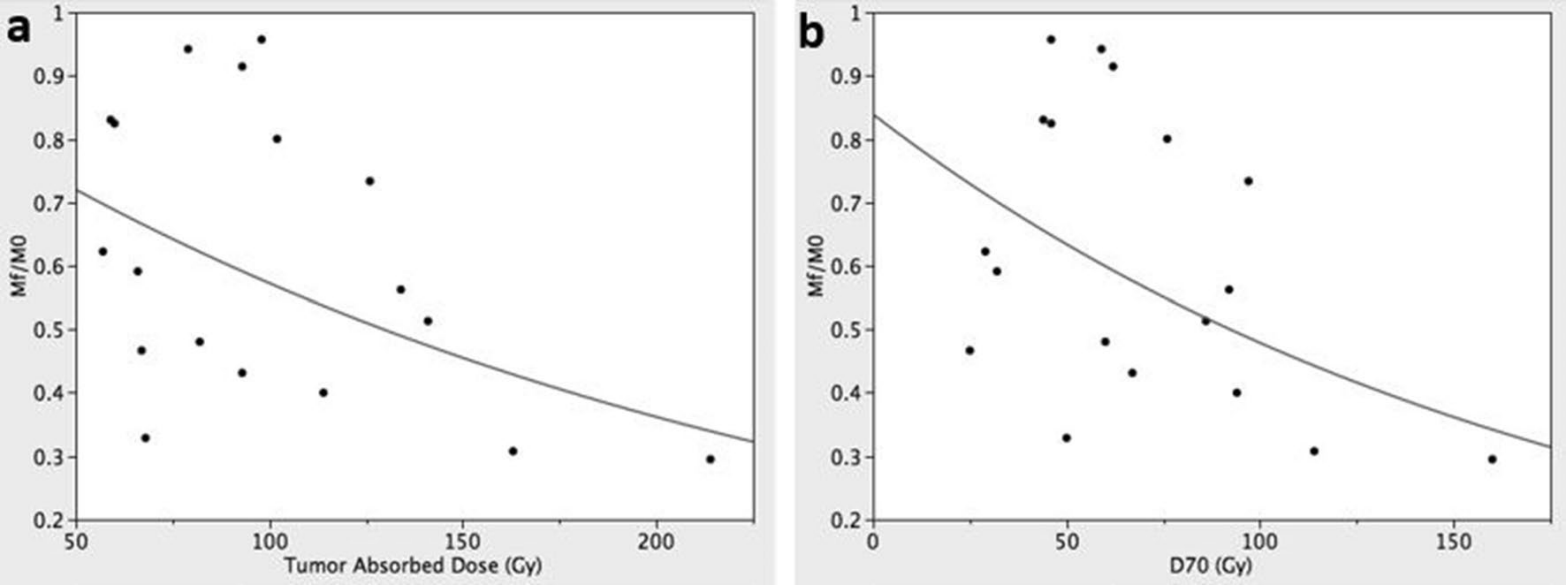
The logarithmic correlation of the residual tumor mass expressed in percentage (M_f_/M_0_) with tumor absorbed dose (r^2^ = 0.24, p < 0.04) (**a**) and D70 (r^2^ = 0.24, p < 0.04) (**b**).

Table 2 summarizes the results of 18 procedures considered for the calculation of the α value. A significant reduction was obtained of the post-treatment tumor mass: M_0_ = 82 g (95% CI 54–200) vs M_f_ = 51 g (95% CI 32–116), p < 0.0001. The time elapsed between SIRT and the achievement of M_f_ was 149 ± 90 days. Tumor average absorbed dose was 93 Gy (95% CI 81–119) and its correlation with the logarithm of the residual tumor mass reduction expressed in percentage [M_f_/M_0_ = 58% (95% CI 51–71)] was statistically significant (r^2^ = 0.24, p < 0.04; Fig. 2a). D70 and V100 were 61 Gy (95% CI 53–85) and 40% (95% CI 29–53), respectively. As for the tumor average absorbed dose, D70 showed a logarithmic correlation with M_f_/M_0_ (r^2^ = 0.24, p < 0.04; Fig. 2b). No significant correlation was observed for V100.

**Table 2:** Procedural details.

Patient (n)	Pre-treatment tumor volume (mL)	Tumor absorbed dose (Gy)	Post-treatment tumor volume (mL)	D70 (Gy)	V100 (%)	α (Gy^−1^)	α_3D_ (Gy^−1^)
1	90	57	56	29	15.1	0.0083	0.0069
2	15	67	7	25	28.3	0.0114	0.0270
3	25	102	20	76	43.2	0.0022	0.0050
4	102	82	49	60	27.7	0.0089	0.0115
5	70	68	23	50	14.3	0.0164	0.0255
5(bis)	86	79	81	59	23.9	0.0008	0.0010
6(r)	76	141	39	86	63.5	0.0047	0.0065
6(l)	16	114	6.4	94	64.1	0.0080	0.0290
7	53	59	44	44	7.3	0.0032	0.0038
8	185	134	104	92	66.8	0.0043	0.0047
9	78	214	23	160	86.2	0.0057	0.0122
10	45	126	33	97	66.9	0.0025	0.0047
11(l)	74	60	61	46	6.9	0.0032	0.0038
11(r)	105	93	96	62	38.2	0.0010	0.0010
12	169	163	52	114	74.7	0.0072	0.0099
13(r + l)	313	93	135	67	42	0.0090	–
14(r + l)	687	66	406	32	16.3	0.0080	–
15(r + l)	93	98	89	46	57.1	0.0004	–

(bis): treatment repeated on the same lesion.

(r): right liver lobe submitted to SIRT in case of bilobar tumors.

(l): left liver lobe submitted to SIRT in case of bilobar tumors.

(r + l): mass-forming lesions with bilobar extension treated with two separated procedure whose data were summed (a volume-weighted average was applied for dose and D70 and a dose-weighted average was applied for V100).

At first follow-up, evaluation of radiological response showed 3 (20%) patients with partial response (PR), 10 (67%) with stable disease (SD) and 2 (13%) with progressive disease (PD). At 3 months, 3 (20%) patients showed PR, 6 (40%) SD and 6 (40%) PD.

Median TTP was 7.3 months (95% CI 3.2–18.0 months). Table 3 shows the results of the univariate and multivariate Cox’s proportional hazards model analysis for some tumor-related continuous variables. Tumor burden, D70 and V100 had no effects on TTP. Multivariate analysis showed a statistically significant negative effect of tumor volume on TTP (p < 0.03) as well as a positive effect of tumor absorbed dose on TTP (p = 0.05).

**Table 3:** Effect of tumor-related continuous variables on time-to-progression.

Variables	Median (95% CI)	Univariate analysis			Multivariate analysis		
		Hazard ratio (95% CI)	Chi-square	P	Hazard ratio (95% CI)	Chi-square	P
Tumor burden (%) (tumor volume/liver volume)	7.6 (95% CI 5.2–12.3)	0.911 (0.770, 1.027)	2.115	0.15			
Tumor volume (mL)	82 (95% CI 54–200)	0.990 (0.975, 0.999)	4.185	0.04	0.986 (0.969, 0.999)	4.841	0.03*
Tumor absorbed dose (Gy)	93 (95% CI 81–119)	1.037 (0.994, 1.082)	2.886	0.09	1.050 (0.998, 1.110)	3.843	0.05*
D70 (Gy)	71 (95% CI 53–85)	1.001 (0.983, 1.017)	0.022	0.88			
V100 (%)	40 (95% CI 29–53)	1.002 (0.975, 1.028)	0.020	0.89			

**Figure 3:**
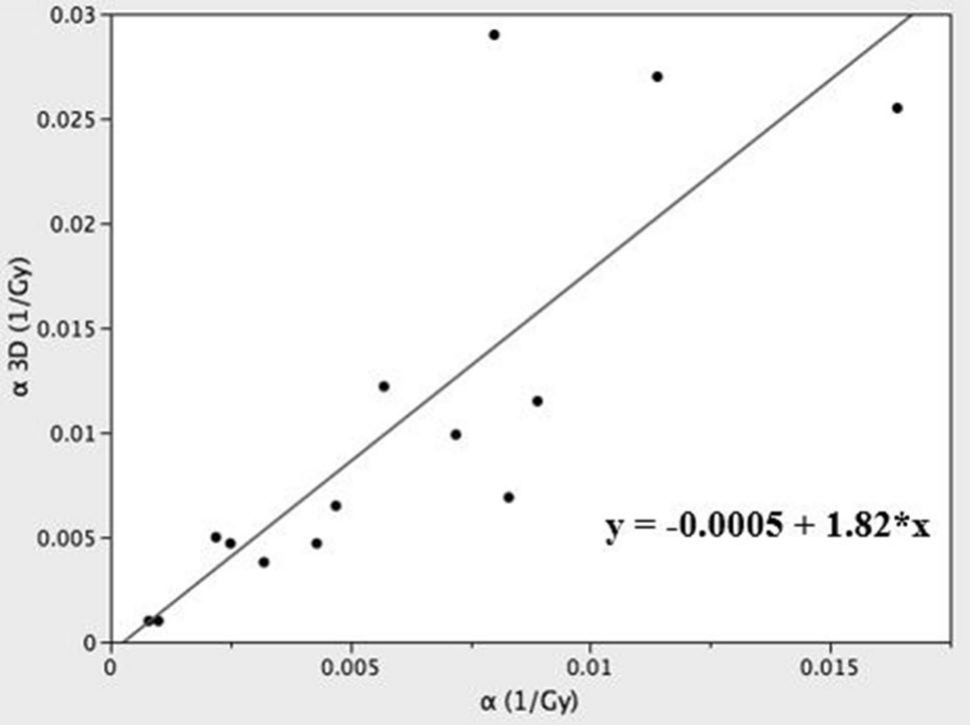
The linear relationship between α and α_3D_ values (r^2^ = 0.69; p = 0.0001).

The α (Eq. 4) and α_3D_ (Eq. 5) values were 0.005 Gy^−1^ (95% CI 0.004–0.008) and 0.007 Gy^−1^ (95% CI 0.005–0.015), respectively. A significant linear relationship (r^2^ = 0.69; p = 0.0001) was obtained between these two parameters showing α_3D_ values higher than α values with a positive slope of about 1.8 and an intercept close to 0 (Fig. 3). No statistically significant difference in α and α_3D_ values was found comparing patients submitted to SIRT alone to patients treated with SIRT plus chemotherapy (Table 4).

**Table 4:** Comparison between patients submitted to SIRT alone and SIRT plus chemotherapy in terms of α and α_3D_ values. No statistically significant differences were found between two groups.

Group of patients	Median (95% CI) of α (number of SIRT procedures)	Median (95% CI)of α_3D_ (number of SIRT procedures)
SIRT alone	0.006 (0.003–0.009) (n = 9)	0.007 (0.009–0.015) (n = 7)
SIRT plus chemotherapy	0.004 (0.003–0.008) (n = 9)	0.006 (0.003–0.019) (n = 8)
P	NS	NS

*NS* not significant.

Following the RECIST 1.1 criteria, radiological PR corresponds to the reduction ≥ 30% of the diameter of the lesion. Considering the approximation of spherical volumes, this corresponds to a final tumor volume M_f_ ≤ 0.34 M_0_.

Using the above-mentioned α_3D_ and α values, the corresponding Equivalent Uniform Dose (EUD_PR_) needed to obtain a radiological PR was:

6 $${EUD}_{PR}\ge -\frac{1}{{\mathrm{\alpha }}_{3D}}\mathrm{ln}\left(\frac{{M}_{f}}{{M}_{0}}\right)=-\frac{1}{0.01}\mathrm{ln}\left(0.34\right)=108 Gy.$$

Similarly, the average dose (D_PR_) needed to obtain a radiological PR resulted to be:

7 $${D}_{PR}\ge -\frac{1}{\mathrm{\alpha }}\mathrm{ln}\left(\frac{{M}_{f}}{{M}_{0}}\right)=-\frac{1}{0.006}\mathrm{ln}\left(0.34\right)=180 Gy.$$

## Discussion

Published studies have reported a wide range of median overall survival after SIRT in ICC (6.1–22 months), probably reflecting the heterogeneity of the selection criteria and treatment protocols^17–29^, as well as the heterogeneous biological behaviour of this relatively rare tumor, whose specific radiosensitivity is largely unknown.

In the present study, the radiosensitivity α of unresectable ICC treated with SIRT was retrospectively analysed, obtaining α values that are substantially in line with previous studies^30^. In particular, Thai et al. reported a mean α value of 0.010 ± 0.001 Gy^−1^^31^ in a series of patients with different liver tumors (including HCC and ICC) treated with external beam radiotherapy.

The α values resulted to be lower than those of α_3D_. Using Eq. (3), the adoption of a lower α translates into a higher average tumor absorbed dose, resulting into a higher probability of obtaining a significant reduction of the final tumor mass (M_f_). Thus, the use of the α value would be preferable to the use of α_3D_ and of the 3D distribution of the tumor absorbed dose (that is even more difficult to assess).

In the setting of HCC, a significant difference between the absorbed dose needed to reach Tumor Control Probability (50%) for glass and for resin microspheres has been reported^11,32^. It probably depends on the intrinsic different microscopic distribution of resin and glass microspheres^10^ due to the different specific activity (activity per microsphere). The differences between these two compounds applies also to ICC. In a series of 64 ICC patients treated with glass microspheres, Bourien et al.^33^ obtained a threshold value of 260 Gy of the tumor absorbed dose to obtain a significant difference in overall survival (28.2 vs 11.4 months), while Levillain et al.^34^ found a significant improvement of overall survival with doses above 86 Gy using resin microspheres (14.9 vs 5.5 months in a population of 58 patients). Thus, the estimated values of tumor radiosensitivity obtained in the present study should be considered specific for resin microspheres.

The relationship between the tumor absorbed dose and the reduction of tumor mass validates the use of the LQ model because it correlates the two variables in a logarithmic way. In the multivariate analysis, TTP was significantly associated to the tumor absorbed dose, supporting the relationship between dose and tumor reduction.

The knowledge of the α value enables personalized dosimetry. In fact, using the Eq. (7), the calculated tumour average absorbed dose (D_PR_ ≥ 180 Gy) needed to obtain a radiological response resulted to be higher than that derived from the standard BSA method (100 Gy in our series). A recent Phase 2 Clinical Trial^35^ enrolled 41 patients with locally advanced ICC treated with chemotherapy combined to SIRT with glass microspheres as first-line treatment obtaining an objective response of 39% at 3 months and a median OS of 22 months. Treatment personalization with the aim to provide at least 205 Gy to the tumor (317 Gy as median dose delivered to the tumor) played one of the main roles in the promising outcomes of this trial.

The main limitation of this retrospective study is represented by the limited number of patients enrolled through a long period of time, with heterogeneous indications and different therapies performed before and after SIRT. As a result, the calculated mean values of α and α_3D_ have a wide standard deviation, probably due to the tumor heterogeneity. No significant differences were observed in the α and α_3D_ values comparing patients treated with SIRT to patients treated with SIRT and chemotherapy; however, the role of concomitant systemic treatments requires further investigation. Moreover, the result of Eq. (7) (D_PR_ ≥ 180 Gy) could be influenced by the tumor approximation of spherical volumes and the use of RECIST criteria that take into account the longest diameter of the lesion to evaluate the response.

Despite the obvious limitations, the study does not aim to draw conclusions on SIRT in patients with unresectable ICC, but it should make a methodological contribution to a greater comprehension of the intrinsic radiosensitivity of ICC in a selected series patients submitted to SIRT with ^90^Y resin microspheres. The knowledge of these radiobiological parameters would enable further advances in the field of personalized dosimetry for SIRT, by calculating the tumor absorbed dose given the tumor specific radiosensitivity and the desired final mass of the treated lesion. Further studies are warranted to investigate how this approach could affect clinical outcomes, in terms of safety, tumor response and survival.
